# Development and validation of the socio-evaluative N-back task to investigate the impact of acute social stress on working memory

**DOI:** 10.1038/s41598-025-22611-0

**Published:** 2025-10-10

**Authors:** Matthias Haucke, Sabrina Golde, Stephan Heinzel

**Affiliations:** 1 Department of Psychiatry and Psychotherapy, Charité – Universitätsmedizin Berlin (Campus Charité Mitte), Charitéplatz 1, 10117 Berlin, Germany; 2 Department of Education and Psychology, Clinical Psychology and Psychotherapy, Freie Universität Berlin, Berlin, Germany; 3 Department of Educational Sciences and Psychology, Clinical and Biological Psychology, Technische Universität Dortmund, Dortmund, Germany

**Keywords:** Social stress, Socio-evaluation, Working memory performance, WM, Electrocardiogram, ECG, Electrodermal activity, EDA, Tonic EDA, RMSSD, Social anxiety disorder, SAD, Social phobia, N-back, Depression, Social anxiety, Human behaviour, Psychology, Health care

## Abstract

Impaired working memory in response to social-evaluative stress might be a central cognitive mechanism underlying the persistence of mental health difficulties such as social anxiety. To investigate this, we developed the Socio-evaluative N-back Task (SENT), which can measure the impact of acute socio-evaluative stress on working memory (WM) performance. Moreover, we tested the SENT in a stratified sample with high (HSA) and low socially anxious (LSA) individuals (HSA = 30, LSA = 27, *N* = 57). Of the highly anxious individuals, thirteen were diagnosed with social anxiety disorder (SAD). The socio-evaluative stress condition, compared to a control condition, increased psychophysiological stress reactions, and reduced WM performance. Moreover, people with low anxiety displayed faster reaction times under socio-evaluative stress compared to a control condition. Conversely, individuals with high social anxiety demonstrated consistent reaction times regardless of whether they encounter a non-evaluative or socio-evaluative situation. This suggests that social anxiety is associated with a lack of differentiation between neutral and socio-evaluative situations. Additionally, this study indicates that the socio-evaluative N-back Task can be used to induce socio-evaluative stress responses while simultaneously measuring working memory and physiological stress responses.

**Supplementary Information:**

The online version contains supplementary material available at 10.1038/s41598-025-22611-0.

## Introduction

 A central cognitive mechanism in the onset and progression of social anxiety may involve the impact of socio-evaluative stress on working memory (WM). WM can be defined as a multicomponent system that updates and manipulates temporarily stored information for higher cognitive functions, such as reasoning or learning^1,2^. This system consists of the subcomponents verbal working memory, visual-spatial working memory, attentional control system and a temporary storage system that modulates and integrates sensory information (“episodic buffer”)^3^. The functioning of these WM systems can be affected by stress through multiple pathways^4,5^. Stress can activate the sympathetic nervous system (SNS) and an increased activity of the hypothalamic–pituitary–adrenal axis (HPA axis)^6^. These processes lead to the release of glucocorticoids from the adrenal cortex (GCs), which can affect prefrontal cortex functioning, such as the capacity to hold and manipulate information temporarily, a core component of working memory (WM)^7,8^. High levels of cortisol can impair the prefrontal cortex (PFC), with noradrenaline influencing PFC function in an inverted U-shaped manner, ranging from fatigue (too little) to alertness (medium) to stress (too much)^9^. This pattern has been reliably demonstrated in both human and animal studies^10^.

**Fig. 1:**
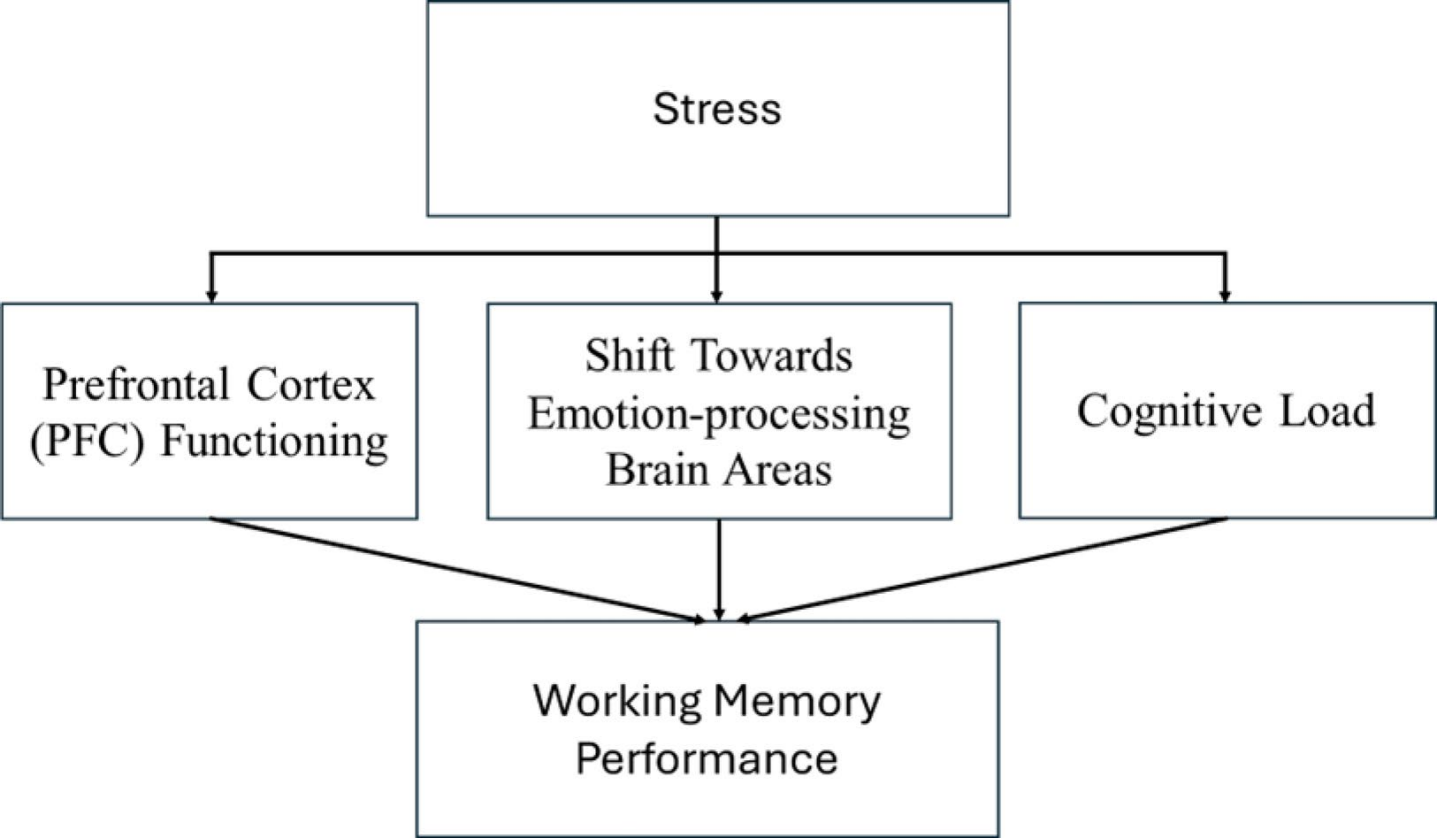
The Pathway of how stress affects working memory performance.

Moreover, stress induces a shift in neural activity toward more emotionally driven brain regions, such as the amygdala, which plays a key role in fear conditioning and the consolidation of emotionally salient information^11^. That is, attention is diverted away from working memory tasks and instead directed toward threat-relevant cues in the environment. Consequently, some studies have reported that stress can enhance the consolidation of rather declarative long-term memories, particularly those with emotional significance^12^. Finally, stress can impair WM by increasing cognitive load through stress-related interference^13^. This includes intrusive thoughts and the intentional suppression of these distressing thoughts, both of which compete for the brain’s limited attentional resources, ultimately decreasing WM performance^14,15^. In sum, stress impairs working memory by disrupting prefrontal cortex (PFC) functioning, shifting neural processes toward brain regions involved in processing emotions, and consuming limited cognitive resources available to the PFC (see Fig. 1). Such stress-related WM disruptions may be associated with distorted or misremembered social experiences, which could play a role in the development and maintenance of social anxiety^16–18^.

Impaired recall of social information may help explain why individuals with social anxiety continue to perceive everyday interactions as threatening, even when evidence suggests otherwise. According to learning theory, a person with SAD should, over time, accumulate evidence that their feared outcomes do not materialize, given the inevitability of social interactions in daily life^19^. Distorted working memory processes might hinder people with SAD to remember, and thus learn from positive social interaction, which could replace overly negative expectations^17^. The Attentional Control Theory^13^ posits that individuals with high social anxiety excessively put resources to process socially threatening information at the cost of task-relevant cognitive processes, which leads to lower task performance. For example, a socially anxious person might have the intrusive and recurrent thought “I will embarrass myself in front of an audience.” when they must perform in a social situation^20^. The resulting negative cognitions then consume attentional resources, while participants attempt to complete a task involving executive functioning. In addition, information that could disconfirm maladaptive beliefs (e.g., noticing that someone is reacting positively to their performance) is less likely to be processed and recalled. In sum, social evaluation is likely to more strongly impair executive control, specifically memory updating, in individuals with elevated social anxiety.

In line with this, previous studies have shown that socially anxious individuals, recall fewer details of positive feedback after holding a presentation^21^ and display a less accurate recall of interpersonal information^22^. Yet, there are only a few studies, with mixed findings, investigating the impact of acute socio-evaluative stress on WM in socially anxious individuals. Two studies using modified version of the Trier Social Stress Task (TSST) found that socio-evaluative stress induction does not lead to lower memory performance in socially anxious individuals^23,24^. Another study, using videotaping as a social stress induction, found decreased memory performance in socially anxious individuals^25^.

The effect of stress on WM is strongly dependent on the intensity, timing and the type of stress^4,12,26^. This paper focuses on the induction of psychosocial stress, as it is particularly relevant for investigating social anxiety, a condition fundamentally characterized by the fear of negative evaluation by others^27^. To successfully evoke psychosocial stress, a laboratory task must^1^ create an uncontrollable situation^2^, create a forced failure context in which one cannot succeed and .^3^ include socio-evaluative threat, for example by having an observer judge the task performance^27^. Prominent paradigms to investigate psychosocial stress induction are the Trier Social Stress Task^28^, Montreal Imaging Stress Task (MIST)^29^ and the ScanSTRESS paradigm^30^. In the following section we will review these paradigms and explain the reasoning for creating the SENT paradigm.

The Trier Social Stress Task^28^, consists of 2 phases: an anticipation phase (10 min) and the task phase (10 min). The task often encompasses to perform a free speech (e.g., job interview) and mental arithmetic in front of an evaluative audience. The TSST has been adapted to measure WM performance; however, WM is measured only after the psychosocial stress induction phase^12,26,31,32^. Consequently, the existing modifications of the TSST paradigm do not allow examining how transient WM processes, such as ongoing memory retrieval and encoding processes, are impacted by acute socio-evaluative stress.

Sociopsychological stress paradigms that allow testing the effects of acute psychophysiological stressors are the ScanSTRESS paradigm^30^ and the Montreal Imaging Stress Task (MIST)^29^. During the MIST, participants are asked to solve mental arithmetic problems (e.g., 27 × 13 − 5) presented on a screen. Socio-evaluative stress is induced by telling participants that they are being evaluated by experimenters or observers (sometimes via camera or live feedback), and by giving negative textual feedback between mental arithmetic tasks (e.g. “Too slow”). Moreover, the task adapts in real time to ensure a high failure rate (e.g., shortening the response time or increasing difficulty). While this method effectively elicits stress responses, it does not include a working memory task. Moreover, it can compromise the reliability of behavioral data, as enforced task failure does not result in comparable working memory performance across individuals (i.e. some participants will have more difficult tasks than others).

During the ScanSTRESS paradigm^30^ participants are required to perform cognitively demanding tasks, a mental rotation and serial subtraction task, under time pressure, all while being observed by a panel of investigators visible via live video feed within the scanner environment. To heighten the social-evaluative threat, the panel delivers standardized negative feedback in real time. Both task difficulty and response speed are dynamically adjusted based on the participant’s performance, ensuring a high rate of failure and a strong sense of uncontrollability. To our awareness, no published study has incorporated a working memory task, such as the N-back, within the ScanSTRESS paradigm. Furthermore, the paradigm’s enforced task failure and dynamic difficulty adjustment compromises the comparability of working memory performance. Finally, both the MIST and ScanSTRESS paradigms allow participants to divert their attention away from the social-evaluative threat, as the threatening cues are either positioned above the task display or, in some cases, not shown at all.

Building on these previous paradigms, we developed the Socio-evaluative N-back Task (SENT), which integrates real-time webcam feedback and adaptive textual and verbal evaluation during a cognitively demanding task. SENT advances previous stress paradigms in three key ways: Firstly, this paradigm allows measuring the impact of acute socio-evaluative stress on ongoing memory retrieval and encoding processes associated with working memory. This is achieved by creating a social stress scenario in which the individual must perform in front of a judgmental audience in real time, rather than performing a cognitive task after stress induction. Secondly, the task prevents participants from diverting their attention away from the social threat. This is accomplished by projecting the task directly in front of the judging audience’s faces, forcing participants to maintain visual contact with the evaluators while completing the task, thereby eliminating the possibility of avoiding eye contact or looking only at the task to reduce discomfort. Finally, this task allows for the collection of comparable working memory performance data during exposure to social-evaluative stress. Unlike adaptive paradigms, the task maintains a consistent level of difficulty, using a demanding working memory task (i.e., 3- N-back task). Furthermore, it provides adaptive feedback by displaying the number of errors or emphasizing when reaction times are too slow, thereby sustaining performance pressure without modifying the task’s level of difficulty.

In sum, this study aims to evaluate the effectiveness of a newly developed social stress paradigm, the Socio-evaluative N-back Task (SENT). Moreover, we examine whether individuals with varying levels of social anxiety exhibit differences in stress response and working memory performance. Task performance is defined as working memory accuracy and response speed. In addition, the study investigates the physiological impact of the SENT task by examining stress-related indicators, including tonic electrodermal activity and heart rate variability. These markers reflect sympathetic and parasympathetic nervous system activity, respectively and are used as indicators of heightened stress^33^. In line with previous studies, we hypothesize that all participants will exhibit heightened tonic electrodermal activity and reduced heart rate variability^28,34^, prolonged reaction times^35^, and reduced memory performance^12,31^, in response to negative social evaluation. In addition, compared to low anxious individuals, we expect that socially anxious individuals will demonstrate heightened physiological stress responses, increased reaction times, and diminished memory performance^25^ in response to socio-evaluative threat.

## Methods

### Sampling and participants

Participants were recruited via bulletins at supermarkets and psychotherapeutic centers, and online advertisements on eBay classifieds and universities’ websites. Participants had to fill in an online pre-questionnaire on the UniPark assessment platform. Participants were recruited if they fall below 13 (i.e., low social anxiety) or above 25 (high social anxiety) on the Social Phobia Inventory (SPIN)^36^. The cut-off scores are based on previous validation studies using the SPIN questionnaire^37,38^. Inclusion criteria encompassed age > 18, ability to consent to the study, and fluent German language skills. Participants were excluded if they suffer from respiratory or cardiac disease, neurological disorders, lifetime-prevalence of Schizophrenia, severe depression, substance use disorder, antisocial and/or borderline personality disorder according to DSM-5^39^ criteria. Moreover, exclusion criteria were pregnancy, heavy speaking impairment, non-correctible vision and hearing impairments, regular drug intake and irregular intake of psychopharmacology. In addition, participants in the control group were excluded if any prior or current diagnosis of mental disorder according to ICD-10/DSM-5 was given. If participants were eligible to participate in the study, they were contacted and we conducted a clinical interview on the German version of SCID-5-CV and SCID-5-PD^40^. A total of 13 participants were diagnosed with social anxiety disorder. All experimental protocols were approved by the Ethics Committee of Freie Universität Berlin (ref: 033/2020), and all methods were carried out in accordance with the relevant guidelines and regulations. We obtained informed consent from all subjects and/or their legal guardian(s) for publication of identifying information/images in an online open-access publication.

### Socio-evaluative N-back task (SENT)

**Fig. 2:**
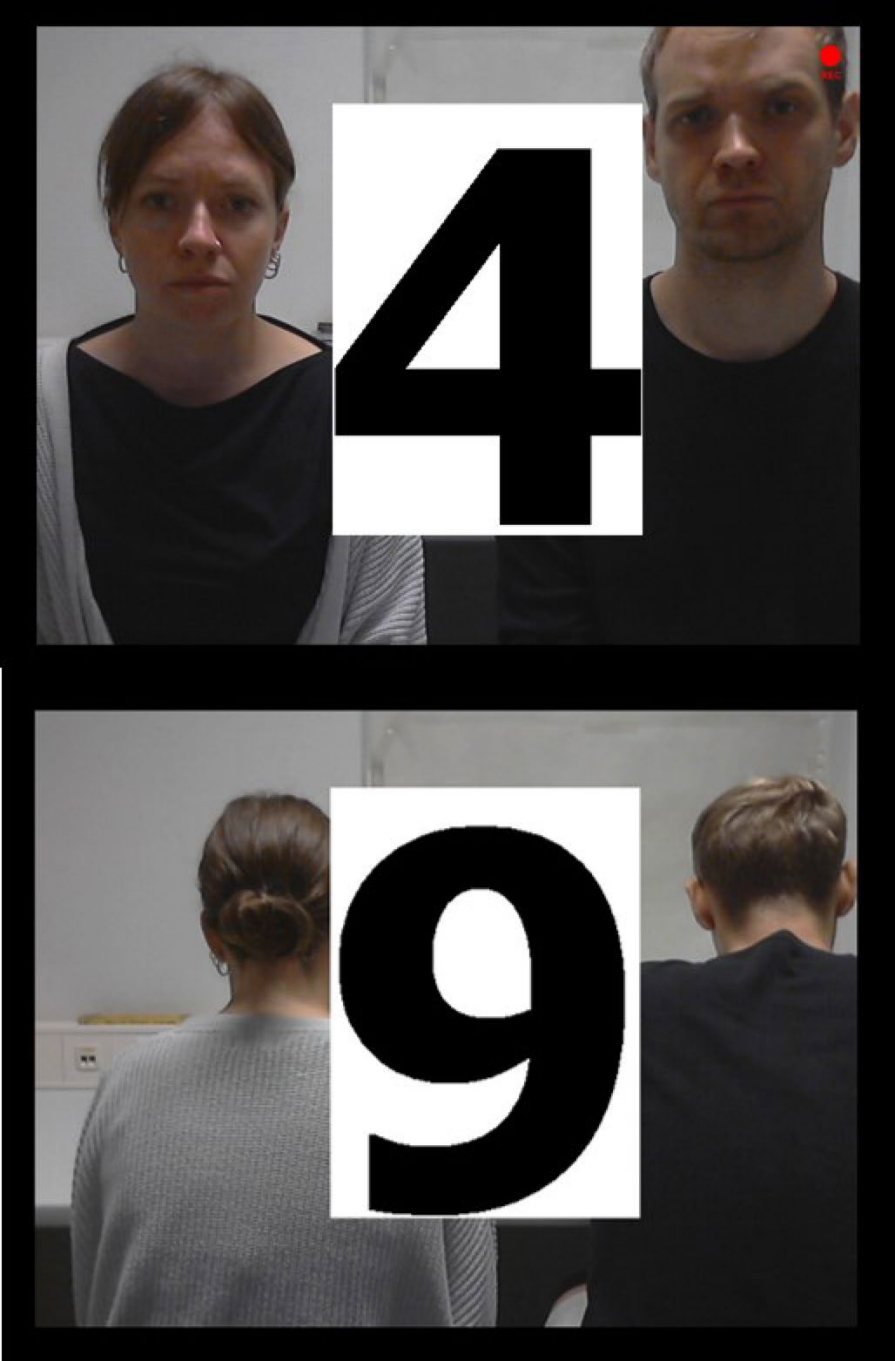
Depiction of the Socio-evaluative N-back Task. During the stress phase, participants were observed by a female and male experimenter (upper picture). During the control phase, the experimenter were faced to the other direction (lower picture).

**Fig. 3:**
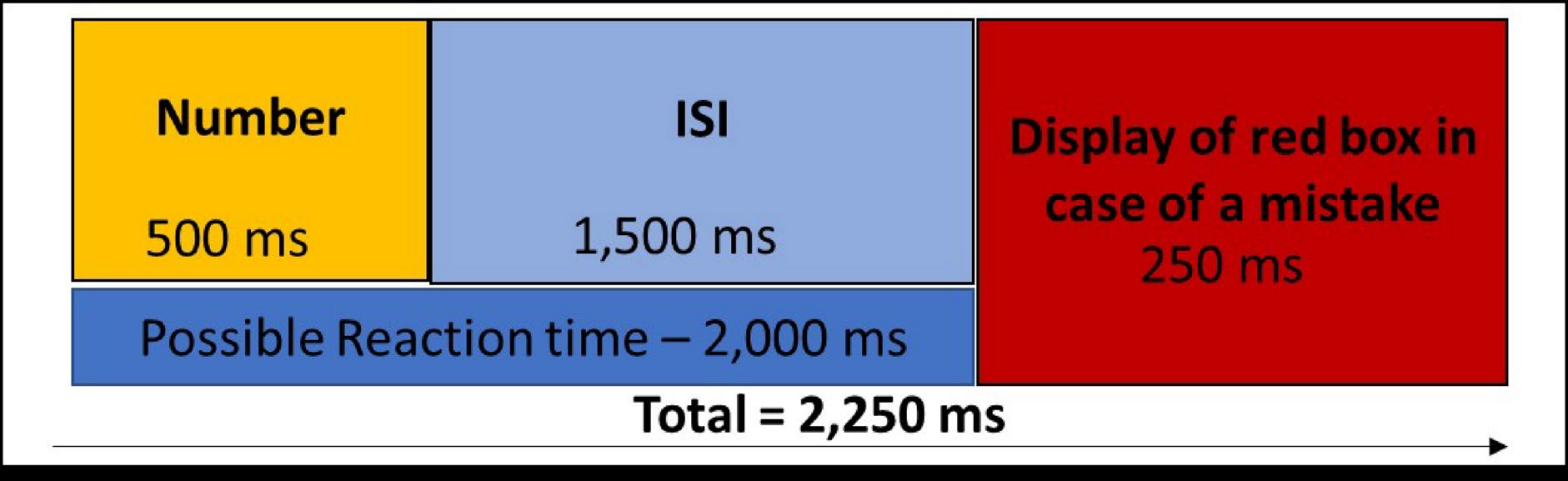
The inter-trial interval, which consisted of number display (500 ms), inter-stimulus interval (ISI) (1500) and an additional 250ms in case a mistake was made. The reaction time window is 2000 ms in total.

**Fig. 4:**
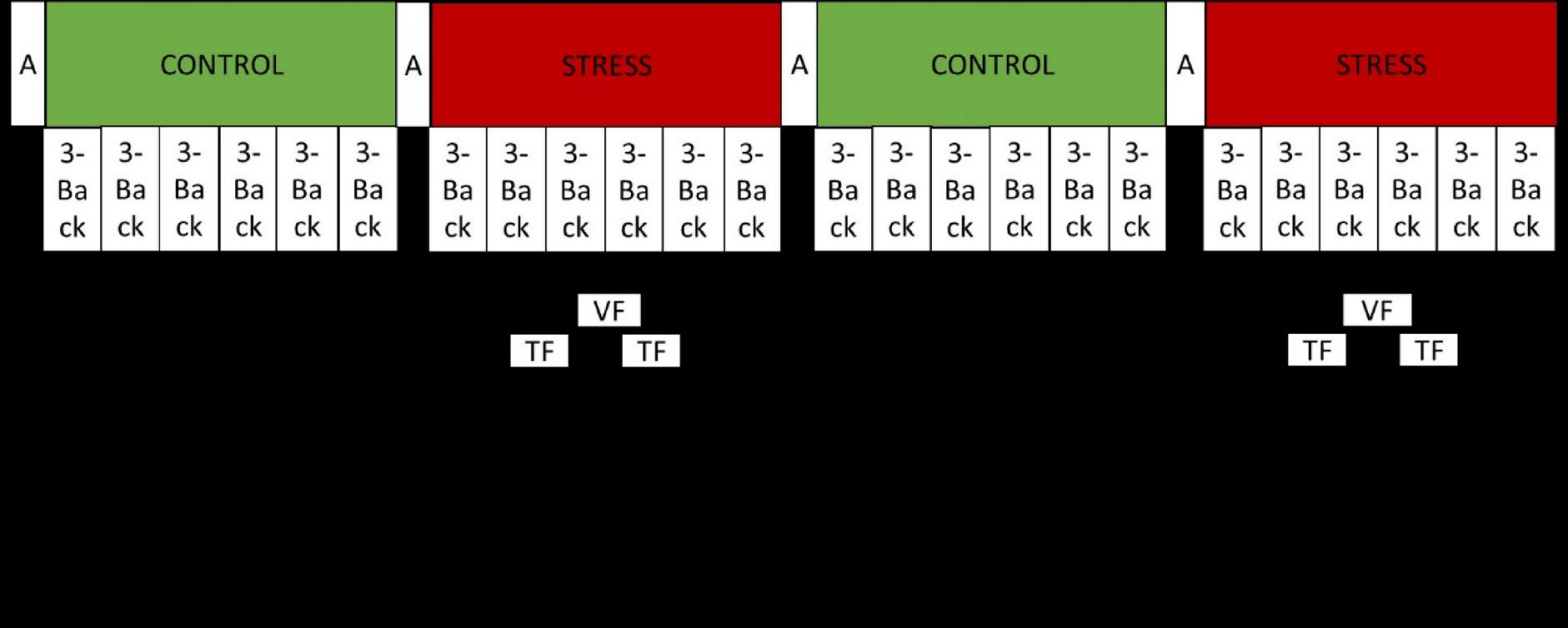
Schematic depiction of the Socio-evaluative N-back task.

During the N-back task, participants continuously observed a screen displaying a live video of one male and one female observer, both of whom had no prior contact to the participants and were seated in a “monitoring room.“. The N-back numbers were then displayed “on top” of the faces of the two observers (see Fig. 2). Therefore, attending to the social evaluative threat could not be avoided if one wants to solve that task. During this task, participants observed a series of 16 briefly presented numbers and decide in each trial (i.e., press a button) if the currently presented number is the same as the number presented *3*-trials ago. Each number was presented for 500 ms, followed by an interstimulus interval of 1500 ms during which the participant could give a response. If participants made a mistake, a red box was displayed surrounding the number for 250ms (see Fig. 3). As shown in Fig. 4, there were 2 randomized control and stress conditions, each condition consists of 6 blocks of N-back tasks, which presented 16 numbers, with 5 randomized targets (pressing button is correct) and 11 randomized non-targets (pressing button is incorrect). The task was programmed using Python (Python Software Foundation, https://www.python.org/) and is freely available on https://osf.io/h29rp/.

In total three experimenters are involved during the task. Person A, the experimenter, explains that the study is a memory performance test examining how effort and concentration relate to physiological responses and whether these can predict performance. Participants are given a chance to practice the N-back task and ask questions. The experimenter also introduces the setup, including the observer room, where a panel of psychologists monitors performance, and the experiment room, where participants complete the task under webcam observation. Persons B and C, a male and a female panel member, introduce themselves, over a webcam from the observer room, as part of the psychological staff responsible for monitoring participants’ performance. They explain the task and tell participants to put in full effort during all sessions. For detailed instructions to the experimenter and the cover story, please refer to the Supplementary Information online.

To ensure comparability of the N-back task performance between subjects, the difficulty of the N-back task stayed even across each N-back blockand all participants. The difficulty level of the N-back task (including factors such as the number of N-backs and inter-stimulus interval between numbers) was set to ensure it was neither overly challenging, which could compromise the validity of the memory performance measure, nor too easy, which may not elicit stress.

Each participant completed the N-back task in both a control and stress condition (see Fig. 4). In stress conditions, any errors, whether false alarms or misses, were visually signaled by a red box appearing around the displayed number. Additionally, participants received both textual and verbal negative feedback. Textual feedback was provided following every second N-back block (each consisting of 16 numbers). When participants committed more than 2 errors during the preceding two blocks a negative text message was displayed, for instance: “You made 3 errors. On average, participants made 2 errors. Specifically, you responded once when a response was not required and missed two required responses.”. Conversely, if participants made no more than 2 errors, the average reaction time minus 80-100ms was automatically calculated and presented as textual negative feedback, such as, “Your reaction time is 621ms, whereas the average participant reacted within 514ms. Your reaction time needs improvement.” This dynamic adjustment of feedback was adopted to enhance the credibility of the negative feedback.

Additionally, participants received verbal negative feedback twice following every third N-back block. A member of the opposite sex, acting as an observer, entered the room and conveyed the following to the participant: (1) expressing dissatisfaction with their performance, (2) emphasizing the necessity for improved performance to ensure data quality, and (3) urging an increase in performance during the subsequent block. In contrast, during the control condition, participants were not observed, and they did not receive any form of negative feedback. Additionally, the observers turned around so that it became clear that they would not be able to see the participant (see Fig. 2).

### Study procedure, measurements and data preparation

After signing an informed consent, participants filled in a questionnaire consisting of demographic questions and the Beck Depression Inventory (BDI-II)^41^. Next, participants had electrodes attached for recording of the electrocardiogram (ECG) and electrodermal activity (EDA). Then, they were seated for a 7-minute resting phase during which they were instructed to look at a fixation cross. Thus, participants had approximately a total of 30-minute resting period before the experiment, to exclude stress caused by arrival to the laboratory. Afterwards, participants were instructed in the N-back task and performed a test trial. The entire study took approximately 55 min to complete.

#### N-back performance

Performance of the N-back task was divided into reaction time and WM accuracy. Reaction time is the time interval between the display of the number and a button press. Memory accuracy was calculated via the following formula:

$$Memory accuracy = (Hits/Possible Hits) - (False Alarms/Possible False Alarms).$$

This was done to penalize participants who press indiscriminately. Participants could correctly press a button in response to targets (= hits), 5 times in each block. Moreover, participants could incorrectly press a button in response to non-targets (= false alarms) 11 times in each N-back block.

### Psychophysiological stress response

####  Tonic electrodermal activity

Electrodermal activity (EDA) was recorded via two sintered Ag/AgCl electrodes attached to the hypothenar of the participant’s non-dominant hand, with a constant voltage of 0.5 V. The signal was recorded using the GSR MR Module by BrainProducts. The incoming raw signal was filtered with a low cutoff time constant (10s) and high cutoff frequency (250 Hz). Skin conductance data was analyzed with the Matlab analysis software Ledalab V3.2.3 (www.ledalab.de). The SCR data were visually inspected and down-sampled to 100 Hz, we applied Butterworth – lowpass filter and the data was separated into phasic and tonic activity via a Decomposition Analysis^42^. This method returns a continuous measure of tonic EDA. This method aims at retrieving the activity properties of the underlying sudomotor nerve activity, avoiding estimation biases produced by overlapping responses. A response window of 36 s was used to cover the N-back trial and the minimum amplitude criterion was set to 0.01 µS, finally the SCR data was z-standardized.

####  Heart rate variability

Electrocardiography (ECG) was recorded via three Ag/AgCl electrodes. Two Ag/AgCl electrodes were attached under the right clavicle and the lowest rib on the left. An additional Ag/AgCl electrode was attached to the left clavicle and served as ground electrode. The signal (1025 Hz) was recorded using the GSR MR Module by BrainProducts. Custom-made Python-scripts (Python Software Foundation, https://www.python.org/) were developed to analyze relevant time frames, including the toolbox BioSSPy^43^, which was used to band-pass filter the data (3 to 45 Hz; finite impulse response filter) and detect R-spikes based on the approach of Hamilton^44^. The detected R-spikes were visually inspected and manually corrected. Moreover, we applied Kamath ectopic beats removal method to the resulting RR-intervals. Using the BioSSPY package, we calculated the root mean square of successive differences between normal heartbeats (RMSSD) for a 36s interval^45^. Thus, RMSSD reflects the beat-to-beat variance in heart rate and is the principal time-domain measure to estimate vagally mediated changes in heart rate variability^46^.

####  Statistical analysis

All statistical analyses were conducted in R (Version 3.5.3, http://cran.r-project.org/). Working memory accuracy and reaction time were averaged for each 3-back block (i.e. 16 trials). That is, we accounted for the multilevel structure of the data by analyzing the effects of stress and control conditions on 24 N-back blocks for each participant. To consider the hierarchical data structure, we build 4 multilevel random intercept models with memory accuracy, reaction time, tonic electrodermal activity and heart rate variability (RMSSD) as outcomes. First level was the individual participant, and second level was their score on the outcome variable. We included stress condition, as well as social anxiety as categorical predictors and their interaction term. Moreover, we included the following control variables (covariates): age, gender, and depressiveness (BDI scores). To determine whether a random intercept or random slope model is more appropriate for our data, we followed a model selection procedure using the Akaike Information Criterion^47^. A more detailed description of the multilevel model, including the random effects can be found online (Supplementary Table S1-S4).

To identify outliers, we visually checked a boxplot of each outcome variable as well employed the z-score method to detect extreme values in normally distributed data (see Supplementary Fig. S1-S4). That is, each value was standardized by calculating its z-score, which measures the number of standard deviations it lies from the mean. Observations with an absolute z-score greater than 3 were considered outliers, as they deviate significantly from the expected range. To assess the robustness of our results, we repeated the analyses after excluding extreme outliers. The results were consistent with those reported in the Results section, suggesting that the observed effects were not attributable to extreme values (see Supplementary Table S5-S8). The analysis script and task can be found on https://osf.io/h29rp/.

## Results

**Fig. 5:**
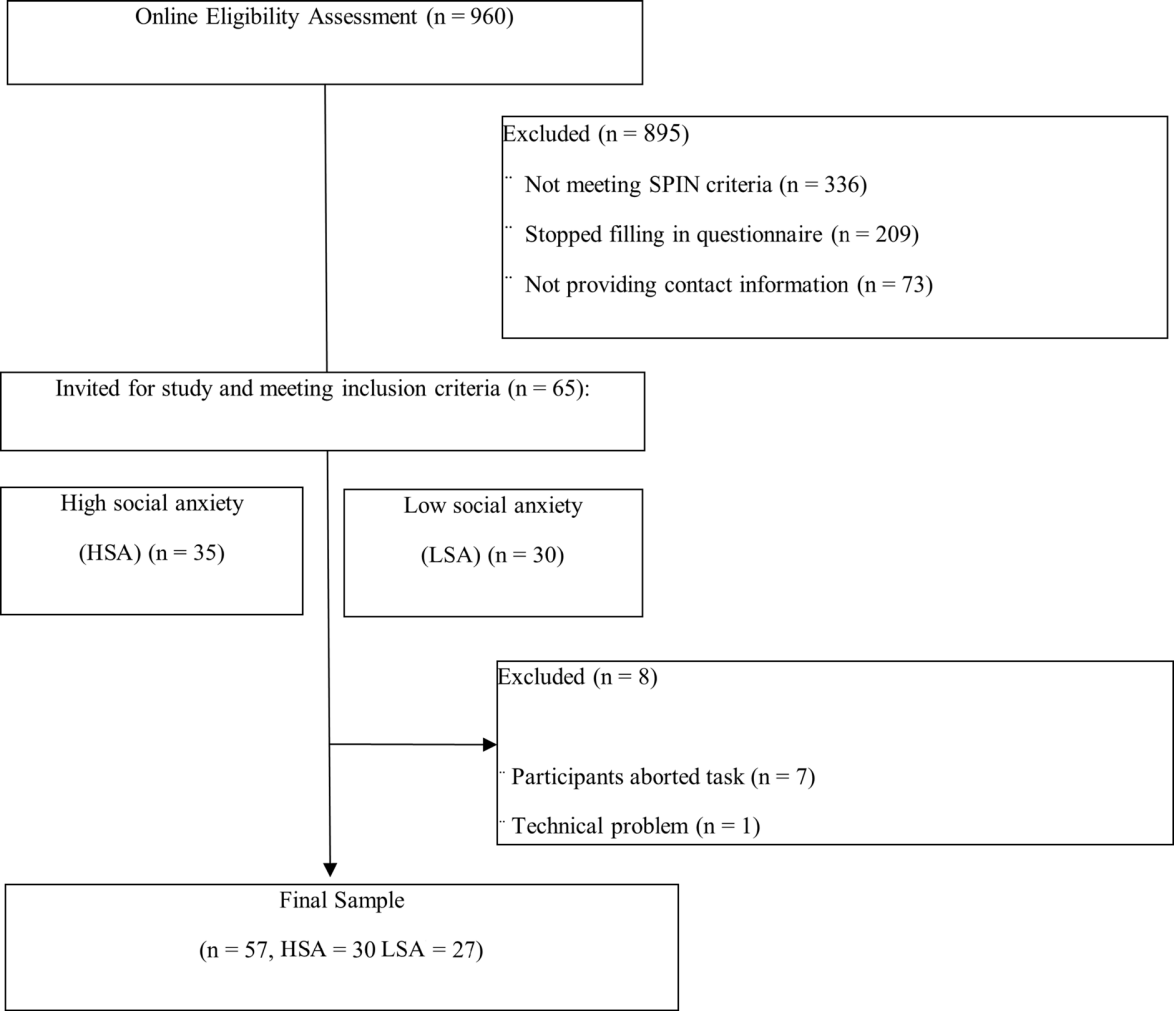
Recruitment flow.

In total, 960 participants initiated the online eligibility questionnaire. A considerable proportion either did not meet the required SPIN criteria (< 13 or > 25; 35%) or discontinued the questionnaire before completion (22%). Of the total 65 invited participants, 7 (3 LSA, 4 HSA; 11% of total sample) stopped participating during the study, because it was too stressful. Due to a technical problem one participant had invalid EDA and ECG data and was subsequently excluded from the analyses. The final sample size was 57. Recruitment flow is shown in Fig. 5, sample characteristics are shown in Table 1.

**Table 1:** Demographics and sample characteristics.

	Age Mean (SD)	Gender Total (percentage)	BDI score Mean (SD)	SPIN score Mean (SD)	SCID diagnosis Total
High social anxiety	31.1 (9.01)	10 male (33%) 20 female (67%)	15.57 (10.79)	34.53 (8.08)	13 Social Anxiety Disorder, 4 Generalized Anxiety Disorder, 4 Major Depression, 1 Obsessive Compulsive Disorder, 3 Panic Disorder, 3 Agoraphobia, 2 Posttraumatic Stress Disorder
Low social anxiety	31.52 (10.61)	13 male (48%) 14 female (52%)	3.85 (4.86)	6.33 (4.21)	None
Total	31.3 (9.72)	23 male (40%) 34 female (60%)	10.02 (10.3)	21.18 (15.62)	13 Social Anxiety Disorder, 4 Generalized Anxiety Disorder, 4 Major Depression, 1 Obsessive Compulsive Disorder, 3 Panic Disorder, 3 Agoraphobia, 2 Posttraumatic Stress Disorder

### Manipulation check

**Fig. 6:**
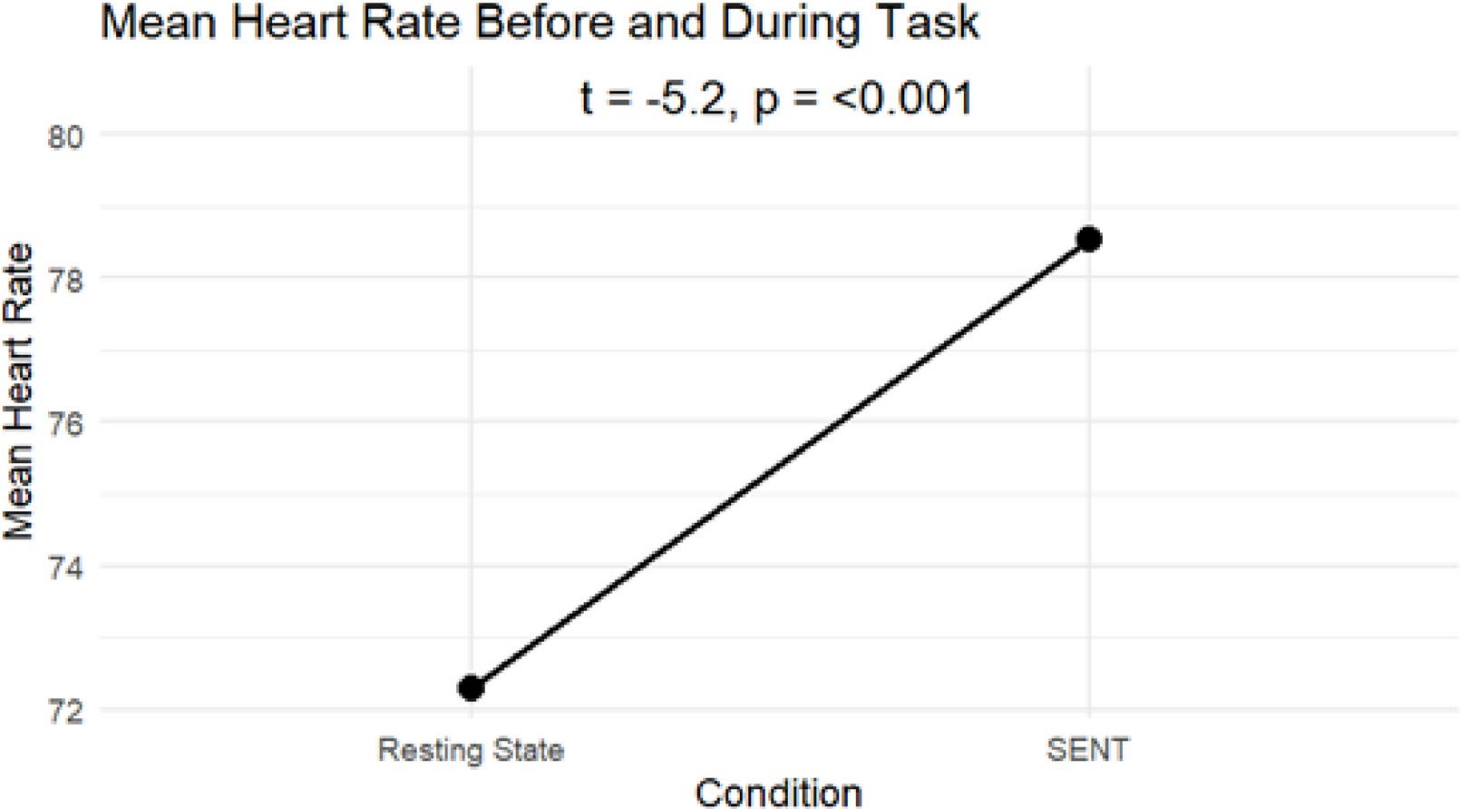
Manipulation check. Comparison of mean heart rate during a 7-minute resting phase and the average heart rate recorded during the Socio-evaluative N-back Task (SENT).

To assess physiological changes before and during the experiment, we compared heart rate during a 7-minute resting state measurement prior to the task with heart rate during the experimental condition. As shown in Fig. 6, there was an increase in heart rate from the resting state to the task phase. A paired samples t-test revealed a statistically significant increase in heart rate from the Resting State (M = 72.3 bpm) to the SENT (M = 78.5 bpm), t(49) = -5.20, *p* < .001. The mean difference was 6.24 bpm, 95% CI [-8.65, -3.83]. This is comparable to the task effect observed in the ScanSTRESS paradigm, which elicited an increase in heart rate of 8.98 bpm during stress conditions^30^.

In general, the feedback provided during the task was accurate, as it was adaptively linked to the participants’ performance. During the post-experiment debriefing, no participant indicated that they were certain the task was deliberately designed to induce stress or that the feedback was false.

### The effects of socio-evaluative stress on reaction time and memory accuracy

**Fig. 7:**
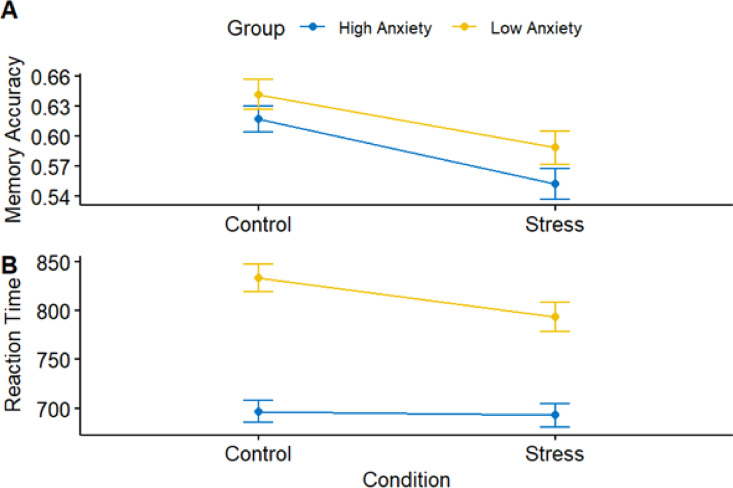
Mean plot of memory accuracy (**A**) and reaction time (**B**) according to the two conditions (Stress/Control), as well as according to the anxiety groups (High/Low Social Anxiety).

Figure 7 illustrates the impact of the between-subject factor social anxiety, as well as the within-subject factor stress and control conditions, on reaction time and memory accuracy.

####  Memory accuracy

Memory accuracy was significantly lower in the stress condition compared to the control condition, b = -0.06, t(1309) = -3.70, *p* < .001. When comparing social anxiety groups, there was no significant difference in memory accuracy between individuals with low and high social anxiety, b = -0.03, t(52) = -0.59, *p* = .559. Additionally, there was no significant interaction between stress condition and social anxiety group, b = 0.01, t(1309) = 0.47, *p* = .64. Among the covariates tested, only depressiveness was significantly associated with memory accuracy, b < 0.01, t(52) = -2.11, *p* = .039. All other covariates were non-significant. See Table 2 for full results.

**Table 2:** Multiple regression results with memory accuracy as the dependent variable.

	Memory accuracy				
	Estimates	Std. error	Statistic	p	df
Predictors					
(Intercept)	0.78	0.07	10.51	**< 0.001**	1309.00
Group [low anxiety]	− 0.03	0.05	− 0.59	0.559	52.00
Condition [stress]	− 0.06	0.02	− 3.70	**< 0.001**	1309.00
Group [low anxiety] * condition [stress]	0.01	0.03	0.47	0.640	1309.00
Covariates					
Depressiveness	− 0.00	0.00	− 2.11	**0.039**	52.00
Age	− 0.00	0.00	− 1.56	0.124	52.00
Gender	0.02	0.04	0.56	0.579	52.00
Random effects					
σ2	0.06				
τ00 VP	0.02				
ICC	0.24				
N VP	57				
Observations	1368				
Marginal R2/Conditional R2	0.057/0.284				

####  Reaction time

Participants with low social anxiety exhibited significantly longer reaction times compared to those with high social anxiety, b = 138.85, t(52) = 2.31, *p* = .025. In contrast, the main effect of stress condition was not significant; participants in the stress condition did not differ significantly in reaction time from those in the control condition, b = -3.17, t(1299) = -0.22, *p* = .787. However, there was a significant interaction between stress condition and social anxiety group, b = -35.80, t(1299) = -2.10, *p* = .036. As shown in Fig. 7, the high social anxiety group responded faster in the stress condition. In the low social anxiety group, reaction times did not significantly differ between the stress and control conditions. Reaction time was not significantly associated with any of the covariates (all p’s > 0.05). See Table 3 for detailed statistics.

**Table 3:** Multiple regression results with reaction time as the dependent variable.

	Reaction time				
	Estimates	Std. error	Statistic	p	df
Predictors					
(Intercept)	633.02	93.93	6.74	**< 0.001**	1299.00
Group [Low Anxiety]	138.85	60.09	2.31	**0.025**	52.00
Condition [Stress]	-3.17	11.75	-0.27	0.787	1299.00
Group *Condition	-35.80	17.06	-2.10	**0.036**	1299.00
Covariates					
BDI	0.51	2.95	0.17	0.864	52.00
Age	2.13	2.56	0.83	0.410	52.00
Gender	-14.80	49.34	-0.30	0.765	52.00
Random effects					
σ2	24486.34				
τ00 VP	31382.11				
N VP	57				
Observations	1358				
Marginal R2/Conditional R2	0.147 / NA				

### The effects of socio-evaluative stress on electrodermal activity and heart rate variability

**Fig. 8:**
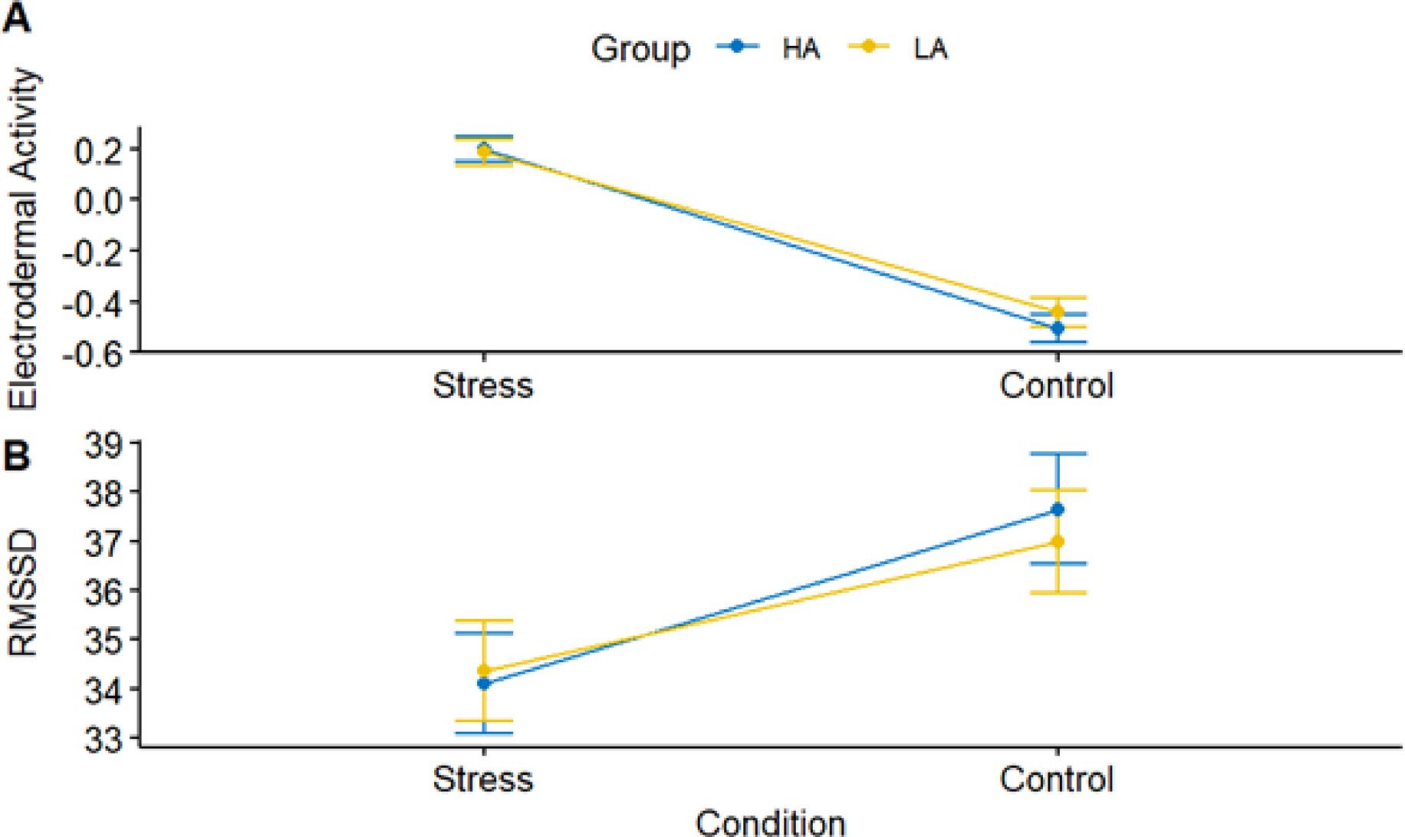
Mean plot of electrodermal activity (**A**) and heart rate variability (RMSSD) (**B**) according to the two conditions (Stress/Control), as well as according to the anxiety groups (High/Low Social Anxiety).

Figure 8 illustrates the impact of the between-subject factor social anxiety, as well as the within-subject factor stress and control conditions, on electrodermal activity and heart rate variability.

#### Tonic electrodermal activity

Tonic electrodermal activity (EDA) was significantly higher in the stress condition compared to the control condition, b = 0.70, t(1337) = 9.73, *p* < .001. Social anxiety group was not significantly associated with tonic EDA, b = 0.04, t(1337) = 0.53, *p* = .559, and there was no significant interaction between stress condition and social anxiety group, b = -0.08, t(1337) = -0.73, *p* = .468. None of the covariates were significantly associated with tonic EDA (all p’s > 0.05). See Table 4 for full results.

**Table 4:** Multiple regression results with tonic EDA as the dependent variable.

	Tonic EDA				
	Estimates	Std. error	Statistic	p	df
Predictors					
(Intercept)	− 0.40	0.11	− 3.64	**< 0.001**	1337.00
Group [Low Anxiety]	0.04	0.08	0.53	0.596	1337.00
Condition [Stress]	0.70	0.07	9.73	**< 0.001**	1337.00
Group *Condition	− 0.08	0.10	− 0.73	0.468	1337.00
Covariates					
BDI	− 0.00	0.00	− 0.46	0.648	1337.00
Age	− 0.00	0.00	− 0.87	0.385	1337.00
Gender	− 0.02	0.05	− 0.35	0.727	1337.00
Observations	1344				
R2/R2 adjusted	0.111/0.107				

####  Heart rate variability (RMSSD)

Root mean square of successive differences (RMSSD) was significantly lower in the stress condition compared to the control condition, b = -3.54, t(1286) = -4.79, *p* < .001. Social anxiety group was not significantly associated with RMSSD, b = -3.54, t(51) = -0.67, *p* = .505. There was also no significant interaction between stress condition and social anxiety group, b = 0.91, t(1286) = 0.85, *p* = .393. None of the covariates were significantly associated with RMSSD (all p’s > 0.05). See Table 5 for detailed results.

**Table 5:** Multiple regression results with tonic EDA as the dependent variable.

	RMSSD				
	Estimates	Std. error	Statistic	p	df
Predictors					
(Intercept)	53.41	8.47	6.31	**< 0.001**	1286.00
Group [LA]	− 3.54	5.27	− 0.67	0.505	51.00
Condition [Stress]	− 3.54	0.74	− 4.79	**< 0.001**	1286.00
Group * Condition	0.91	1.06	0.85	0.393	1286.00
Covariates					
BDI	− 0.30	0.27	− 1.12	0.266	51.00
Age	− 0.38	0.23	− 1.67	0.101	51.00
Gender	0.68	4.37	0.16	0.877	51.00
Random effects					
σ2	94.60				
τ00 VP	247.92				
N VP	56				
Observations	1344				
Marginal R2 / Conditional R2	0.211 / NA				

### Exploratory analyses

In an exploratory analysis, we used the social anxiety disorder (SAD) classification as a categorical predictor, rather than relying on the high/low socially anxious group classification. Our study encompasses 13 participants diagnosed with SAD, and 44 individuals who did not meet the criteria. Using WM performance, reaction time and RMSSD as an outcome, analysis revealed the same pattern as described above, except for tonic EDA (see Supplementary Table S9−S12). Therefore, we present the findings of the analysis with the outcome variable tonic EDA.

**Fig. 9:**
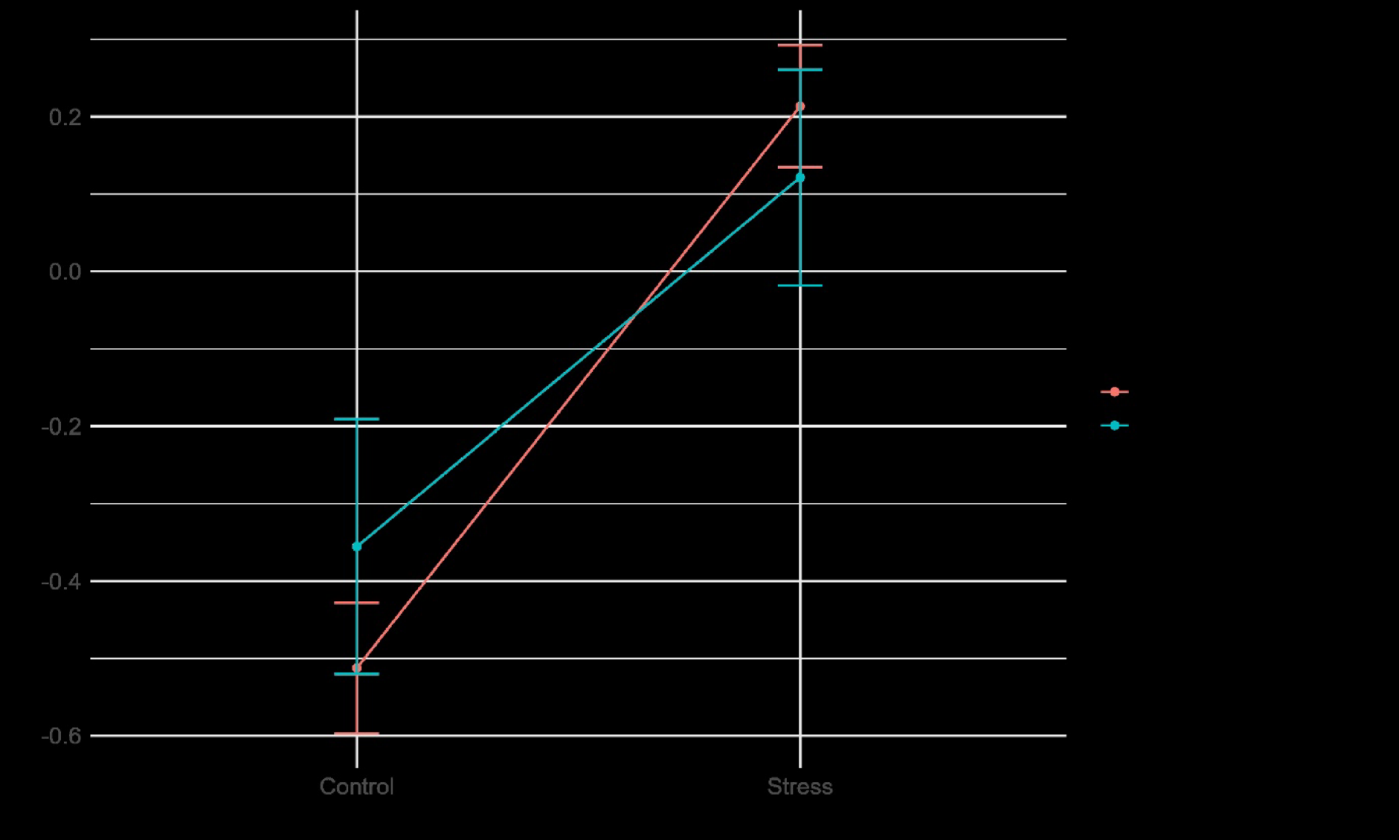
Mean plot of interaction between Stress Condition and Social Anxiety Disorder group. The x-axis shows the Control and Stress Condition. The y-axis is the z-standardized tonic EDA. The red line is the no SAD group, whereas the blue line shows the SAD group.

Tonic electrodermal activity (EDA) was significantly higher in the stress condition compared to the control condition, b = 0.73, t(1337) = 12.23, *p* < .001. Individuals with social anxiety disorder (SAD) exhibited significantly higher overall tonic EDA than those without SAD, b = 0.20, t(1337) = 2.07, *p* = .038. There was a significant interaction between stress condition and SAD diagnosis, b = -0.25, t(1337) = -2.02, *p* = .043. As illustrated in Fig. 9, individuals without SAD displayed a steeper increase in tonic EDA during the stress condition than those with SAD. Additionally, the SAD group exhibited a higher level of tonic EDA in the control condition. None of the covariates were significantly associated with tonic EDA (all p’s > 0.05).

## Discussion

Although impaired working memory performance under socio-evaluative stress may serve as a key cognitive mechanism underlying mental health difficulties such as social anxiety^17^, there is a lack of paradigms that can effectively induce social stress while simultaneously measuring working memory performance. This study sets out to test a newly developed socio-evaluative paradigm, called the Socio-evaluative N-back Task (SENT). We found that the stress condition compared to the control condition successfully increased psychophysiological stress responses and decreased WM accuracy. Contrary to our hypothesis, participants with low social anxiety did not exhibit reduced working memory accuracy or slower reaction times during the stress condition. Instead, they demonstrated faster reaction times in the control condition compared to the control group. Thus, individuals with high social anxiety consistently exhibit fast reaction times, regardless of exposure to socio-evaluative threat. In sum, the SENT can be used to investigate the effects of acute socio-evaluative stress on psychophysiological stress responses on working memory.

We found that the social evaluative stress condition resulted in reduced working memory (WM) accuracy compared to the control condition. These findings support the previously established link between psychosocial stress and working memory^4,12,26^. In addition, our study supports the previously found link between socio-evaluative stress and diminished parasympathetic nervous system activity, indicated by decreased heart rate variability^46^. In addition, our results suggests that socio-evaluative stress can activate the sympathetic nervous system, indexed by increased electrodermal activity^34^. In sum, during the socio-evaluative stress condition, compared to a control condition, all participants displayed decreased WM performance, as well as heightened physiological stress responses.

Contrary to our hypothesis, we found that individuals with high social anxiety consistently responded quickly and maintained memory accuracy, regardless of whether they were under social evaluation or not. Moreover, contrary to our hypothesis, individuals with social anxiety did not exhibit heightened physiological arousal in response to the socio-evaluative stress condition. This suggests that individuals with low social anxiety may not distinguish between control and stress conditions, as their responses to the control condition closely resemble those under social evaluation. Similarly, exploratory analyses indicate that individuals diagnosed with social anxiety disorder (SAD) exhibited heightened physiological arousal, as measured by electrodermal activity (EDA), even during the control task. This effect might lead to a “ceiling effect”, as people with social anxiety already showed a higher baseline physiological arousal, which led to a less steep increase of physiological reaction during the control phase. This finding is in line with previous findings, showing a higher baseline physiological stress reaction and flattened increase in people with social anxiety^48,49^.

As a result, the difference in arousal between the control and stress conditions was less pronounced compared to individuals without SAD. Over time, this lack of differentiation could place an additional burden on cognitive resources of socially anxious individuals, ultimately leading to reduced working memory performance, given that they are limited^15^. According to the “Yerkes–Dodson law”, performance increases with physiological or mental arousal, but only up to a point. When levels of arousal become too high, performance decreases^50^. The underlying reasons for this should be explored in future studies; however, one possibility is that individuals with social anxiety may be more motivated to please the experimenter, which could lead to heightened performance even during the control condition. Less differentiation between socio-evaluative threats from neutral contexts may, over time, contribute to cognitive strain and could explain the mixed evidence on WM impairments in individuals with high social anxiety^18,24,51–54^.

Furthermore, this inconsistency in findings could be attributed to laboratory setups that evaluate WM only after stress-induction tasks, potentially diminishing the effects of stress on working memory. In contrast to other socio-evaluative stress paradigms such as the Trier Social Stress Task with N-back^12^, ScanSTRESS^30^, or the Montreal Imaging Stress Task^29^, the Socio-evaluative N-back Task (SENT) enables the assessment of the impact of acute socio-evaluative stress on ongoing working memory (WM) performance. Moreover, this task cannot be solved when participants shift their attention away from the socio-evaluative situation. Finally, this task enables the collection of comparable working memory (WM) performance data, as the task’s difficulty remains constant throughout trials and across all participants.

### Limitations and future studies

In this study, we primarily investigated individuals with subclinical social anxiety, only 43% of the highly socially anxious individuals were diagnosed with SAD. The findings of the exploratory analysis must be interpreted with caution due to marked group size differences. In particular, small sample sizes in physiological measures increase the risk of regression to the mean, which may artificially drive observed interactions^55^. To understand the impact of socio-evaluative stress on working memory in SAD, future research should aim to investigate these effects in a larger sample with individuals diagnosed with SAD.

Additionally, our use of an artificial laboratory setting might potentially limit the external validity of our findings. Moreover, we found that depressiveness decreased working memory accuracy. Prior research suggests that working memory impairment is a cognitive feature of major depressive disorder (MDD)^6,56,57^. Notably, major depressive disorder and social anxiety disorder commonly co-occur, with prevalence rates ranging from 15% to 33%^58,59^. To advance our understanding, future investigations should distinguish more precisely between the impacts of depression and anxiety on working memory impairments in SAD^60,61^.

Another limitation of this study is that we cannot fully distinguish between the effects of socio-evaluative stress and distraction caused by visual cues, such as facial stimuli. Therefore, it is possible that the observed task effects were driven less by stress per se and more by competing cognitive demands arising from emotional processing. Previous research suggests that negative facial expressions (e.g., fear, anger) are particularly disruptive to working memory, likely due to their threat-related salience^62^. In addition, this task includes multiple forms of negative evaluation, such as textual and verbal feedback, all of which are likely to influence physiological stress markers and task performance.

Future studies could disentangle these effects by systematically varying the presence of facial, textual, or no evaluative stimuli across experimental conditions, allowing for direct comparison of their individual impacts. Additionally, incorporating eye-tracking methods^63^ could help clarify the attentional mechanisms underlying responses to emotional cues during the SENT paradigm. A key strength of the SENT is its ability to assess how socio-evaluative stress influences both working memory and physiological responses in real time. This makes it a valuable tool for capturing dynamic, event-related changes as they unfold. Future studies could build on this by examining how specific task events elicit stress responses and whether participants show distinct learning curves or adaptation patterns throughout the task.

## Conclusion

This study set out to test a newly developed laboratory socio-evaluative stress paradigm, called the Socio-evaluative N-back Task (SENT). We found that the socio-evaluative stress condition, compared to a control condition, increased psychophysiological stress response (i.e., increased tonic electrodermal activity and decreased heart rate variability), and decreased WM performance. In contrast to individuals with low social anxiety, highly socially anxious individuals show no difference in reaction time between the control and socio-evaluative stress conditions. This indicates that socially anxious individuals tend to not differentiate socio-evaluative threat from a neutral situation. In sum, we can conclude that the Socio-evaluative N-back Task (SENT) has the potential to induce socio-evaluative stress responses while simultaneously measuring working memory.

## Supplementary Information

Below is the link to the electronic supplementary material.

Supplementary Material 1

## Data Availability

The datasets used and/or analysed during the current study are available from the corresponding author on reasonable request.

## References

[1] Baddeley, A. Working memory: looking back and looking forward. *Nat. Rev. Neurosci.* **4** (10), 829–839 (2003). 14523382 10.1038/nrn1201

[2] Chai, W. J., Abd Hamid, A. I. & Abdullah, J. M. Working memory from the psychological and neurosciences perspectives: a review. *Front. Psychol.* **9**, 401 (2018). 29636715 10.3389/fpsyg.2018.00401 PMC5881171

[3] Baddeley, A. Working memory. *Curr. Biol.* **20** (4), R136–R40 (2010). 20178752 10.1016/j.cub.2009.12.014

[4] Lupien, S. J., Gillin, C. J. & Hauger, R. L. Working memory is more sensitive than declarative memory to the acute effects of corticosteroids: A dose–response study in humans. *Behav. Neurosci.* **113** (3), 420 (1999). 10443770 10.1037//0735-7044.113.3.420

[5] Al’Absi, M., Hugdahl, K. & Lovallo, W. R. Adrenocortical stress responses and altered working memory performance. *Psychophysiology* **39** (1), 95–99 (2002). 12206301 10.1017/S0048577202001543

[6] De Kloet, E. R., Joëls, M. & Holsboer, F. Stress and the brain: from adaptation to disease. *Nat. Rev. Neurosci.* **6** (6), 463–475 (2005). 15891777 10.1038/nrn1683

[7] Ellis, K. A. & Nathan, P. J. The Pharmacology of human working memory. *Int. J. Neuropsychopharmacol.* **4** (3), 299–313 (2001). 11602036 10.1017/S1461145701002541

[8] Robbins, T. W. Chemistry of the mind: neurochemical modulation of prefrontal cortical function. *J. Comp. Neurol.* **493** (1), 140–146 (2005). 16254988 10.1002/cne.20717

[9] Arnsten, A. F. & Li, B-M. Neurobiology of executive functions: catecholamine influences on prefrontal cortical functions. *Biol. Psychiatry*. **57** (11), 1377–1384 (2005). 15950011 10.1016/j.biopsych.2004.08.019

[10] Arnsten, A. F. Stress signalling pathways that impair prefrontal cortex structure and function. *Nat. Rev. Neurosci.* **10** (6), 410–422 (2009). 19455173 10.1038/nrn2648 PMC2907136

[11] Roozendaal, B. Stress and memory: opposing effects of glucocorticoids on memory consolidation and memory retrieval. *Neurobiol. Learn. Mem.* **78** (3), 578–595 (2002). 12559837 10.1006/nlme.2002.4080

[12] Schoofs, D., Preuß, D. & Wolf, O. T. Psychosocial stress induces working memory impairments in an n-back paradigm. *Psychoneuroendocrinology* **33** (5), 643–653 (2008). 18359168 10.1016/j.psyneuen.2008.02.004

[13] Eysenck, M. W., Derakshan, N., Santos, R. & Calvo, M. G. Anxiety and cognitive performance: attentional control theory. *Emotion* **7** (2), 336 (2007). 17516812 10.1037/1528-3542.7.2.336

[14] Stawski, R. S., Sliwinski, M. J. & Smyth, J. M. Stress-related cognitive interference predicts cognitive function in old age. *Psychol. Aging*. **21** (3), 535 (2006). 16953715 10.1037/0882-7974.21.3.535 PMC2957652

[15] Klein, K. & Boals, A. The relationship of life event stress and working memory capacity. *Appl. Cogn. Psychology: Official J. Soc. Appl. Res. Memory Cognition*. **15** (5), 565–579 (2001).

[16] Burt, D. B., Zembar, M. J. & Niederehe, G. Depression and memory impairment: a meta-analysis of the association, its pattern, and specificity. *Psychol. Bull.* **117** (2), 285 (1995). 7724692 10.1037/0033-2909.117.2.285

[17] Clark, D. M. Wells AJSpD, assessment, treatment. *Cogn. model. Social Phobia*. **41** (68), 00022–00023 (1995).

[18] Waechter, S. et al. Working memory capacity in social anxiety disorder: revisiting prior conclusions. *J. Abnorm. Psychol.* **127** (3), 276 (2018). 29672089 10.1037/abn0000341

[19] Seligman, M. E. *Competing Theories of Panic* 321–329 (Routledge, 2013).

[20] Hackmann, A., Surawy, C. & Clark, D. M. Seeing yourself through others’eyes: & of spontaneously occurring images in social phobia. A study. *Behav. Cogn. Psychother.* **26** (1), 3–12 (1998).

[21] Glazier, B. L. & Alden, L. E. Social anxiety and biased recall of positive information: it’s not the content, it’s the Valence. *Behav. Ther.* **48** (4), 533–543 (2017). 28577588 10.1016/j.beth.2016.08.001

[22] Hope, D. A., Heimberg, R. G. & Klein, J. F. Social anxiety and the recall of interpersonal information (1990).

[23] Espín, L., Marquina, M., Hidalgo, V., Salvador, A. & Gómez-Amor, J. No effects of psychosocial stress on memory retrieval in non-treated young students with generalized social phobia. *Psychoneuroendocrinology* **73**, 51–62 (2016). 27464065 10.1016/j.psyneuen.2016.07.211

[24] Maresh, E. L., Teachman, B. A. & Coan, J. A. Are you watching me? Interacting effects of fear of negative evaluation and social context on cognitive performance. *J. Experimental Psychopathol.* **8** (3), 303–319 (2017).

[25] Graver, C. J. & White, P. M. Neuropsychological effects of stress on social phobia with and without comorbid depression. *Behav. Res. Ther.* **45** (6), 1193–1206 (2007). 17010931 10.1016/j.brat.2006.08.002

[26] Oei, N. Y. et al. Glucocorticoids decrease hippocampal and prefrontal activation during declarative memory retrieval in young men. *Brain Imaging Behav.* **1** (1), 31–41 (2007). 19946603 10.1007/s11682-007-9003-2 PMC2780685

[27] Stein, M. B. & Stein, D. J. Social anxiety disorder. *Lancet* **371** (9618), 1115–1125 (2008). 18374843 10.1016/S0140-6736(08)60488-2

[28] Kirschbaum, C., Pirke, K-M. & Hellhammer, D. H. The ‘Trier social stress Test’–a tool for investigating Psychobiological stress responses in a laboratory setting. *Neuropsychobiology* **28** (1–2), 76–81 (1993). 8255414 10.1159/000119004

[29] Dedovic, K. et al. The Montreal imaging stress task: using functional imaging to investigate the effects of perceiving and processing psychosocial stress in the human brain. *J. Psychiatry Neurosci.* **30** (5), 319 (2005). 16151536 PMC1197276

[30] Streit, F. et al. A functional variant in the neuropeptide S receptor 1 gene moderates the influence of urban upbringing on stress processing in the amygdala. *Stress* **17** (4), 352–361 (2014). 24800784 10.3109/10253890.2014.921903

[31] Jiang, C. & Rau, P-L-P. Working memory performance impaired after exposure to acute social stress: the evidence comes from erps. *Neurosci. Lett.* **658**, 137–141 (2017). 28851617 10.1016/j.neulet.2017.08.054

[32] Luers, P., Schloeffel, M. & Prüssner, J. C. Working memory performance under stress. *Exp. Psychol.* (2020). 10.1027/1618-3169/a000484 32729402

[33] Bosch, O. J., Nair, H. P., Ahern, T. H., Neumann, I. D. & Young, L. J. The CRF system mediates increased passive stress-coping behavior following the loss of a bonded partner in a monogamous rodent. *Neuropsychopharmacology* **34** (6), 1406–1415 (2009). 18923404 10.1038/npp.2008.154 PMC2669698

[34] Reinhardt, T., Schmahl, C., Wüst, S. & Bohus, M. Salivary cortisol, heart rate, electrodermal activity and subjective stress responses to the Mannheim multicomponent stress test (MMST). *Psychiatry Res.* **198** (1), 106–111 (2012). 22397919 10.1016/j.psychres.2011.12.009

[35] Debener, S. et al. Trial-by-trial coupling of concurrent electroencephalogram and functional magnetic resonance imaging identifies the dynamics of performance monitoring. *J. Neurosci.* **25** (50), 11730–11737 (2005). 16354931 10.1523/JNEUROSCI.3286-05.2005 PMC6726024

[36] Connor, K. M. et al. Psychometric properties of the social phobia inventory (SPIN): new self-rating scale. **176** (4), 379–386 (2000). 10.1192/bjp.176.4.379 10827888

[37] Badra, M. et al. The association between ruminative thinking and negative interpretation bias in social anxiety. *Cogn. Emot.* **31** (6), 1234–1242 (2017). 27279528 10.1080/02699931.2016.1193477

[38] Sosic, Z., Gieler, U. & Stangier, U. Screening for social phobia in medical in-and outpatients with the German version of the social phobia inventory (SPIN). *J. Anxiety Disord.* **22** (5), 849–859 (2008). 17923381 10.1016/j.janxdis.2007.08.011

[39] Association, A. P. *Diagnostic and Statistical Manual of Mental Disorders (DSM-5®)* (American Psychiatric Pub, 2013).

[40] Beesdo-Baum, K., Zaudig, M. & Wittchen, H. *Strukturiertes Klinisches Interview für DSM-5 [The Structured Clinical Interview for DSM-5-Clinician Version (SCID-5-CV. 1* (Hogrefe, 2019).

[41] Beck, A. T., Steer, R. A. & Brown, G. K. J. S. A. Beck depression inventory-II. **78** (2), 490–498 (1996).

[42] Benedek, M. & Kaernbach, C. Decomposition of skin conductance data by means of nonnegative Deconvolution. *Psychophysiology* **47** (4), 647–658 (2010). 20230512 10.1111/j.1469-8986.2009.00972.x PMC2904901

[43] Bota, P., Silva, R., Carreiras, C. & Fred, A. Plácido Da Silva H. BioSPPy: A python toolbox for physiological signal processing. *SoftwareX* **26**, 101712 (2024).

[44] Ajdaraga, E. & Gusev, M. (eds) *Analysis of Sampling Frequency and Resolution in ECG Signals. 2017 25th Telecommunication Forum (TELFOR)* (IEEE, 2017).

[45] Baek, H. J., Cho, C-H., Cho, J. & Woo, J-M. Reliability of ultra-short-term analysis as a surrogate of standard 5-min analysis of heart rate variability. *Telemedicine e-Health*. **21** (5), 404–414 (2015). 25807067 10.1089/tmj.2014.0104

[46] Shaffer, F., McCraty, R. & Zerr, C. L. A healthy heart is not a metronome: an integrative review of the heart’s anatomy and heart rate variability. *Front. Psychol.* **5**, 1040 (2014). 25324790 10.3389/fpsyg.2014.01040 PMC4179748

[47] Akaike, H. A new look at the statistical model identification. *IEEE Trans. Autom. Control*. **19** (6), 716–723 (1974).

[48] Fiksdal, A. et al. Associations between symptoms of depression and anxiety and cortisol responses to and recovery from acute stress. *Psychoneuroendocrinology* **102**, 44–52 (2019). 30513499 10.1016/j.psyneuen.2018.11.035 PMC6420396

[49] Klumbies, E., Braeuer, D., Hoyer, J. & Kirschbaum, C. The reaction to social stress in social phobia: discordance between physiological and subjective parameters. *Plos One*. **9** (8), e105670 (2014). 25153526 10.1371/journal.pone.0105670 PMC4143269

[50] Calabrese, E. J. Converging concepts: adaptive response, preconditioning, and the Yerkes–Dodson law are manifestations of hormesis. *Ageing Res. Rev.* **7** (1), 8–20 (2008). 17768095 10.1016/j.arr.2007.07.001

[51] Berggren, N. & Anxiety Anxiety and apprehension in visual working memory performance: no change to capacity, but poorer distractor filtering. *Stress Coping* **33** (3), 299–310 (2020). 10.1080/10615806.2020.1736899 32126798

[52] Edelmann, R. J. & Baker, S. R. Self-reported and actual physiological responses in social phobia. *Br. J. Clin. Psychol.* **41** (1), 1–14 (2002). 11931674 10.1348/014466502163732

[53] Grossman, P., Wilhelm, F. H., Kawachi, I. & Sparrow, D. Gender differences in Psychophysiological responses to speech stress among older social phobics: congruence and incongruence between self-evaluative and cardiovascular reactions. *Psychosom. Med.* **63** (5), 765–777 (2001). 11573025 10.1097/00006842-200109000-00010

[54] Mauss, I., Wilhelm, F. & Gross, J. Is there less to social anxiety than Meets the eye? Emotion experience, expression, and bodily responding. *Cogn. Emot.* **18** (5), 631–642 (2004).

[55] Barnett, A. G., Van Der Pols, J. C. & Dobson, A. J. Regression to the mean: what it is and how to deal with it. *Int. J. Epidemiol.* **34** (1), 215–220 (2005). 15333621 10.1093/ije/dyh299

[56] Rose, E. & Ebmeier, K. Pattern of impaired working memory during major depression. *J. Affect. Disord.* **90** (2–3), 149–161 (2006). 16364451 10.1016/j.jad.2005.11.003

[57] Vasic, N., Walter, H., Sambataro, F. & Wolf, R. Aberrant functional connectivity of dorsolateral prefrontal and cingulate networks in patients with major depression during working memory processing. *Psychol. Med.* **39** (6), 977–987 (2009). 18845009 10.1017/S0033291708004443

[58] Belzer, K. & Schneier, F. R. Comorbidity of anxiety and depressive disorders: issues in conceptualization, assessment, and treatment. *J. Psychiatric Pract.*. **10** (5), 296–306 (2004). 15361744 10.1097/00131746-200409000-00003

[59] Zimmerman, M., Chelminski, I. & McDermut, W. Major depressive disorder and axis I diagnostic comorbidity. *J. Clin. Psychiatry*. **63** (3), 187–193 (2002). 11926716 10.4088/jcp.v63n0303

[60] Amir, N. & Bomyea, J. Working memory capacity in generalized social phobia. *J. Abnorm. Psychol.* **120** (2), 504 (2011). 21381805 10.1037/a0022849 PMC3229920

[61] Turner, S. M., Beidel, D. C. & Larkin, K. T. Situational determinants of social anxiety in clinic and nonclinic samples: physiological and cognitive correlates. *J. Consult. Clin. Psychol.* **54** (4), 523 (1986). 3745606 10.1037//0022-006x.54.4.523

[62] Dolcos, F. & McCarthy, G. Brain systems mediating cognitive interference by emotional distraction. *J. Neurosci.* **26** (7), 2072–2079 (2006). 16481440 10.1523/JNEUROSCI.5042-05.2006 PMC6674921

[63] Yousefi, M. S., Reisi, F., Daliri, M. R. & Shalchyan, V. Stress detection using eye tracking data: an evaluation of full parameters. *IEEE Access.* **10**, 118941–118952 (2022).

