# Supplementary material for: Development and validation of the socio-evaluative N-back task to investigate the impact of acute social stress on working memory
Source: Sci Rep. 2025 Oct 10;15:35408. doi: 10.1038/s41598-025-22611-0 (PMC12514150; doi:10.1038/s41598-025-22611-0)

**Supplementary Information**

**Instructions and cover story**

**Person A** (Experimenter):

**1. before the SST - experimenter instruction to participants**

- “This experiment is a memory performance test. “

- “We investigate the relationship of effort and concentration with skin conductance and heart rate.”

- “Now you can practice the task once and ask questions if necessary”

Experimenter goes back to the test room with participant, start the exercise task.

**2. exercise task before real SST**

If participants have at least one sequence of 16 numbers with 2 or less errors, continue with experiment

**3. before the SST- guided tour of the observation room**

- Guide through the observation room, show the control monitor, point out the webcam in the experiment room (webcam on!)

“Now I show you the rooms, here is the observer room”

“The panel consist of psychologist who will observe your performance to make sure that you are doing the task correctly so that we can analyze the data”

“Here is the experiment room where you sit”

“We will observe you over this webcam”

**Panel (Person B and C):**

**4. more detailed instructions from the panel via loudspeaker- first contact to participant**

- “ Hello, I am Mr..., this is Mrs... we are psychological staff in this project, we have to make sure that you do the task correctly”

- “There will be 2 types of sessions. Sessions in which we observe you via a webcam directed on you and evaluate your performance.”

- “And there are sessions in which you are not observed and not assessed. In these rounds you can practice the task again to improve your performance. Your own webcam is switched off in these rounds”.

- “During the rounds we evaluate, an incorrect answer is indicated by the appearance of a red frame around the picture”

- “After every 2^nd^ round you will receive feedback on your performance”

- “ Durin the unrated rounds you are not observed and receive no feedback”

- “Speed AND correctness of the answer count as performance”

- “Please make every effort in ALL rounds. Even in the unrated rounds.”

Behavior during stress condition:

Evaluated run: Look directly into the camera! Shake head/frown after error message, do not smile, look serious. Remain credible.

After 3 trials, go into the room and say the following:

If, too many mistakes

- “You have made too many mistakes (second round: still too many mistakes)”

- “If your performance does not improve, this will jeopardize the usability of the results”

(second round: “Please try really hard again, otherwise we won't be able to use your data”)

- “Please put more effort and concentration in the next rounds”

If, hardly any mistakes

- “You react too slowly (second round: still too slow)”

- “If your performance does not improve, this will jeopardize the usability of the results”

(second round: “Please make a real effort again, otherwise we will not be able to use your data”)

- “Please put more effort and concentration in the next rounds.”

Unrated pass:

- Don't look at the camera or screen,

- Turn away from the monitor, read something (take your cell phone/book with you)

**6. debriefing (AFTER EMA study)**

-This is a stress test, therefore we gave you exaggerated negative feedback

-Your answers are influenced by stress; this does not reflect your true memory ability.

-Please do not talk about content with other participants

**Supplementary Tables**

**Supplementary Table S1**. Multilevel Modell building process. Multilevel regression with memory performance as an outcome. To determine whether a random intercept or random slope model is more appropriate for our data, we followed a model selection procedure using the Akaike Information Criterion (Akaike, 1974). We stopped adding random effects when the AIC did not further decrease.

| **Model** | AIC |
| --- | --- |
| Fixed intercept | 379.62 |
| **Random Intercept, Fixed Slopes** | **59.24** |
| Random Intercept, Random Slope “Anxiety Group” | 61.96 |

**Supplementary Table S2.** Multilevel Modell building process. Multilevel regression with reaction time as an outcome. To determine whether a random intercept or random slope model is more appropriate for our data, we followed a model selection procedure using the Akaike Information Criterion (Akaike, 1974). We stopped adding random effects when the AIC did not further decrease.

| **Model** | AIC |
| --- | --- |
| Fixed intercept | 18799.91 |
| **Random Intercept, Fixed Slopes** | **17792.31** |
| Random Intercept, Random Slope “Anxiety Group” | 17793.64 |

**Supplementary Table S3.** Multilevel Modell building process. Multilevel regression with tonic EDA as outcome. To determine whether a random intercept or random slope model is more appropriate for our data, we followed a model selection procedure using the Akaike Information Criterion (Akaike, 1974). We stopped adding random effects when the AIC did not further decrease.

| **Model** | AIC |
| --- | --- |
| **Fixed intercept** | **3846.24** |
| Random intercept | 3848.24 |

**Supplementary Table S4.** Multilevel Modell building process. Multilevel regression with RMSSD as outcome. To determine whether a random intercept or random slope model is more appropriate for our data, we followed a model selection procedure using the Akaike Information Criterion (Akaike, 1974). We stopped adding random effects when the AIC did not further decrease.

| **Model** | AIC |
| --- | --- |
| **Fixed intercept** | **11757.96** |
| Random intercept | 10206.97 |
| **Random intercept, fixed slopes** | **10179.59** |
| Random intercept , frandom slope “Social anxiety group” | 10183.38 |

**Supplementary Table S5.** Results of multilevel regression analysis (with outlier removal) predicting working memory performance. Z-standardization identified 10 working memory performance scores that exceeded three standard deviations from the mean. These outliers were excluded from the analysis.

|  | **WM Performance** | | | | |
| --- | --- | --- | --- | --- | --- |
| Predictors | Estimates | std. Error | Statistic | p | df |
| (Intercept) | 0.76 | 0.07 | 10.86 | **<0.001** | 1299.00 |
| Group [Low Anxiety] | -0.02 | 0.05 | -0.45 | 0.655 | 52.00 |
| Condition [Stress] | -0.06 | 0.02 | -3.40 | **0.001** | 1299.00 |
| BDI | -0.00 | 0.00 | -2.07 | **0.043** | 52.00 |
| Age | -0.00 | 0.00 | -1.49 | 0.141 | 52.00 |
| Gender | 0.02 | 0.04 | 0.59 | 0.557 | 52.00 |
| Group [Low Anxiety] × Condition [Stress] | 0.01 | 0.03 | 0.43 | 0.670 | 1299.00 |
| Random Effects | | | | | |
| σ^2^ | 0.05 | | | | |
| τ_00_ _VP_ | 0.02 | | | | |
| ICC | 0.23 | | | | |
| N _VP_ | 57 | | | | |
| Observations | 1358 | | | | |
| Marginal R^2^ / Conditional R^2^ | 0.053 / 0.269 | | | | |

**Supplementary Table S6.** Results of multilevel regression analysis (with outlier removal) predicting reaction time. Z-standardization identified 9 reaction time scores that exceeded three standard deviations from the mean. These outliers were excluded from the analysis.

|  | **Reaction Time** | | | | |
| --- | --- | --- | --- | --- | --- |
| Predictors | Estimates | std. Error | Statistic | p | df |
| (Intercept) | 624.59 | 91.93 | 6.79 | **<0.001** | 1290.00 |
| Group [Low Anxiety] | 139.39 | 58.76 | 2.37 | **0.021** | 52.00 |
| Condition [Stress] | -6.98 | 10.91 | -0.64 | 0.523 | 1290.00 |
| BDI | 0.62 | 2.89 | 0.21 | 0.831 | 52.00 |
| Age | 2.33 | 2.51 | 0.93 | 0.357 | 52.00 |
| Gender | -16.83 | 48.30 | -0.35 | 0.729 | 52.00 |
| Group [Low Anxiety] × Condition [Stress] | -38.38 | 15.87 | -2.42 | **0.016** | 1290.00 |
| Random Effects | | | | | |
| σ^2^ | 21036.57 | | | | |
| τ_00_ _VP_ | 30167.30 | | | | |
| ICC | 0.59 | | | | |
| N _VP_ | 57 | | | | |
| Observations | 1349 | | | | |
| Marginal R^2^ / Conditional R^2^ | 0.078 / 0.621 | | | | |

**Supplementary Table S7.** Results of multilevel regression analysis (with outlier removal) predicting tonic electrodermal activity. Z-standardization identified 2 reaction time scores that exceeded three standard deviations from the mean. These outliers were excluded from the analysis.

|  | **tonic EDA** | | | | |
| --- | --- | --- | --- | --- | --- |
| Predictors | Estimates | std. Error | Statistic | p | df |
| (Intercept) | -0.39 | 0.11 | -3.64 | **<0.001** | 1284.00 |
| Group [Low Anxiety] | 0.05 | 0.08 | 0.56 | 0.579 | 51.00 |
| Condition [Stress] | 0.72 | 0.07 | 9.97 | **<0.001** | 1284.00 |
| BDI | -0.00 | 0.00 | -0.40 | 0.687 | 51.00 |
| Age | -0.00 | 0.00 | -1.08 | 0.286 | 51.00 |
| Gender | -0.02 | 0.05 | -0.32 | 0.750 | 51.00 |
| Group [Low Anxiety] × Condition [Stress] | -0.08 | 0.10 | -0.73 | 0.468 | 1284.00 |
| Random Effects | | | | | |
| σ^2^ | 0.89 | | | | |
| τ_00_ _VP_ | 0.00 | | | | |
| N _VP_ | 56 | | | | |
| Observations | 1342 | | | | |
| Marginal R^2^ / Conditional R^2^ | 0.116 / NA | | | | |

**Supplementary Table S8.** Results of multilevel regression analysis (with outlier removal) predicting RMSSD. Z-standardization identified 13 RMSSD scores that exceeded three standard deviations from the mean. These outliers were excluded from the analysis.

|  | **RMSSD** | | | | |
| --- | --- | --- | --- | --- | --- |
| Predictors | Estimates | std. Error | Statistic | p | df |
| (Intercept) | 52.37 | 8.14 | 6.43 | **<0.001** | 1273.00 |
| Group [LA] | -2.54 | 5.07 | -0.50 | 0.619 | 51.00 |
| Condition [Stress] | -3.25 | 0.69 | -4.70 | **<0.001** | 1273.00 |
| BDI | -0.26 | 0.26 | -1.01 | 0.317 | 51.00 |
| Age | -0.39 | 0.22 | -1.75 | 0.086 | 51.00 |
| Gender | 0.38 | 4.20 | 0.09 | 0.928 | 51.00 |
| Group [LA] × Condition y [1] | 0.60 | 0.99 | 0.60 | 0.546 | 1273.00 |
| Random Effects | | | | | |
| σ^2^ | 81.53 | | | | |
| τ_00_ _VP_ | 229.34 | | | | |
| ICC | 0.74 | | | | |
| N _VP_ | 56 | | | | |
| Observations | 1331 | | | | |
| Marginal R^2^ / Conditional R^2^ | 0.069 / 0.756 | | | | |

**Supplementary Table S9.** Exploratory multilevel regression with memory performance as an outcome. Model with random intercept (AIC = 59.51).

|  | Memory Performance | | | | |
| --- | --- | --- | --- | --- | --- |
| Predictors | Estimates | std. Error | Statistic | p | df |
| (Intercept) | 0.75 | 0.07 | 10.86 | <0.001 | 1309.00 |
| SAD [Yes] | 0.02 | 0.06 | 0.36 | 0.719 | 52.00 |
| Condition [Stress] | -0.06 | 0.01 | -4.14 | <0.001 | 1309.00 |
| BDI | -0.00 | 0.00 | -2.08 | 0.042 | 52.00 |
| Age | -0.00 | 0.00 | -1.55 | 0.127 | 52.00 |
| Gender | 0.02 | 0.04 | 0.63 | 0.531 | 52.00 |
| SAD * Condition | 0.00 | 0.03 | 0.11 | 0.911 | 1309.00 |
| **Random Effects** | | | | | |
| σ2 | 0.06 | | | | |
| τ00 VP | 0.02 | | | | |
| ICC | 0.24 | | | | |
| N VP | 57 | | | | |
| Observations | 1368 | | | | |
| Marginal R2 |  | | | | |

**Supplementary Table S10.** Exploratory multilevel regression with reaction time as an outcome. Model with random intercept (AIC = 17795.14).

|  | Reaction Time | | | | |
| --- | --- | --- | --- | --- | --- |
| Predictors | Estimates | std. Error | Statistic | p | df |
| (Intercept) | 723.92 | 91.61 | 7.90 | <0.001 | 1299.00 |
| SAD [Yes] | -44.24 | 72.46 | -0.61 | 0.544 | 52.00 |
| Condition [Stress] | -30.97 | 9.68 | -3.20 | 0.001 | 1299.00 |
| BDI | -2.48 | 3.02 | -0.82 | 0.414 | 52.00 |
| Age | 2.81 | 2.67 | 1.05 | 0.298 | 52.00 |
| Gender | -25.52 | 50.91 | -0.50 | 0.618 | 52.00 |
| SAD * Condition | 47.73 | 20.33 | 2.35 | 0.019 | 1299.00 |
| **Random Effects** | | | | | |
| σ2 | 24465.64 | | | | |
| τ00 VP | 33696.17 | | | | |
| N VP | 57 | | | | |
| Observations | 1358 | | | | |
| Marginal R2 |  | | | | |

**Supplementary Table S11.** Exploratory multilevel regression with tonic EDA as an outcome. No multilevel structure assumed (AIC = 3846.24).

|  | tonic EDA | | | | |
| --- | --- | --- | --- | --- | --- |
| Predictors | Estimates | std. Error | Statistic | p | df |
| (Intercept) | -0.42 | 0.10 | -4.20 | <0.001 | 1337.00 |
| SAD [Yes] | 0.20 | 0.10 | 2.07 | 0.038 | 1337.00 |
| Condition [Stress] | 0.73 | 0.06 | 12.23 | <0.001 | 1337.00 |
| BDI | -0.00 | 0.00 | -1.11 | 0.267 | 1337.00 |
| Age | -0.00 | 0.00 | -0.65 | 0.518 | 1337.00 |
| Gender | -0.02 | 0.05 | -0.30 | 0.763 | 1337.00 |
| SAD * Condition | -0.25 | 0.12 | -2.02 | 0.043 | 1337.00 |
| Observations | 1344 | | | | |
| R2 |  | | | | |

**Supplementary Table S12.** Exploratory multilevel regression with RMSSD as an outcome. Random intercept model (AIC = 10180.65)

|  | RMSSD | | | | |
| --- | --- | --- | --- | --- | --- |
| Predictors | Estimates | std. Error | Statistic | p | df |
| (Intercept) | 51.20 | 8.07 | 6.35 | <0.001 | 1286.00 |
| SAD [Yes] | 0.45 | 6.24 | 0.07 | 0.942 | 51.00 |
| Condition [Stress] | -3.07 | 0.60 | -5.11 | <0.001 | 1286.00 |
| BDI | -0.22 | 0.26 | -0.84 | 0.405 | 51.00 |
| Age | -0.40 | 0.23 | -1.72 | 0.091 | 51.00 |
| Gender | 0.96 | 4.37 | 0.22 | 0.827 | 51.00 |
| SAD * Condition | -0.17 | 1.30 | -0.13 | 0.893 | 1286.00 |
| **Random Effects** | | | | | |
| σ2 | 94.65 | | | | |
| τ00 VP | 249.46 | | | | |
| N VP | 56 | | | | |
| Observations | 1344 | | | | |
| Marginal R2 | 0.201 | | | | |

**Supplementary Figures**

**Supplementary Figure S1.** Boxplot of Working Memory (WM) Performance: Sensitivity Analysis After Outlier Removal


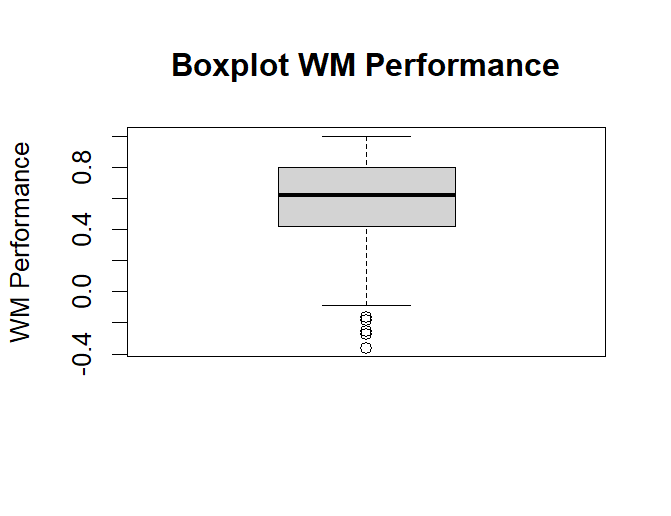


**Supplementary Figure S2.** Boxplot of Reaction Time during the N-Back Task: Sensitivity Analysis After Outlier Removal


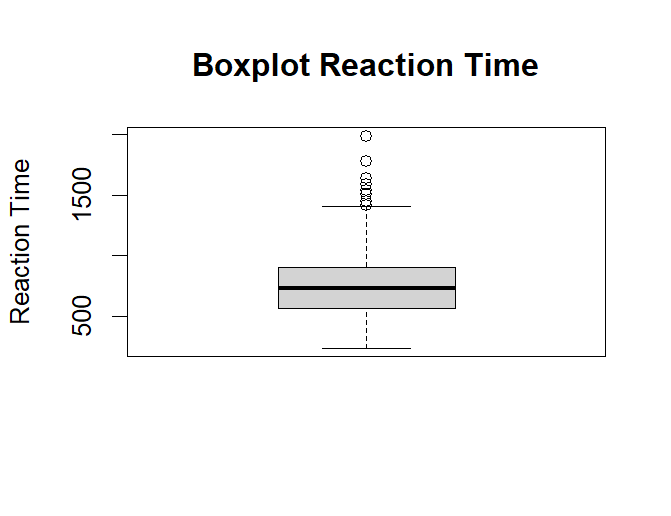


**Supplementary Figure S3.** Boxplot of tonic electrodermal activity (EDA): Sensitivity Analysis After Outlier Removal


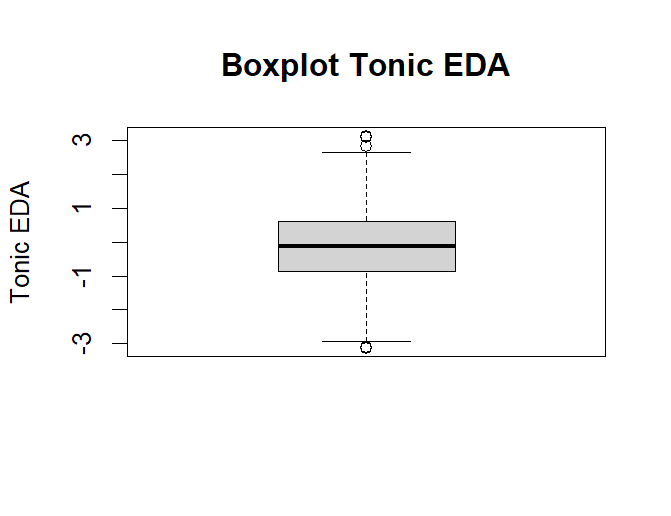


**Supplementary Figure S4.** Boxplot of Root Mean Square of Successive Differences (RMSSD) derived from ecg data: Sensitivity Analysis After Outlier Removal


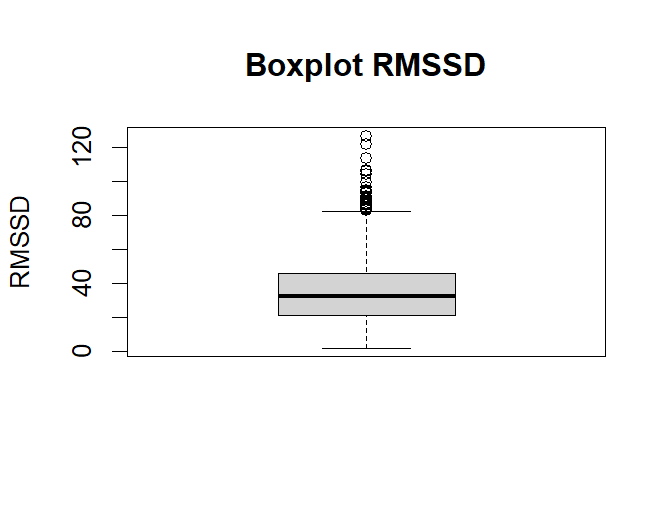

Supplement: Supplementary file 1 — Supplementary Material 1 [file 41598_2025_22611_MOESM1_ESM.docx]
